# Role of Prophylactic Aggressive Hydration to Prevent Post-endoscopic Retrograde Cholangiopancreatography Pancreatitis: A Randomized Controlled Trial

**DOI:** 10.7759/cureus.110675

**Published:** 2026-06-11

**Authors:** Mohammad Omar Yousofzai, Marwah Ijaz, Shaik Taha, M Osama Ghaffar, M Kamran Siddique, Muhammad Irfan Jamil, Jahangeer Ahmed, Ammar Noor, Adeel Ahmed, Tayyaba Arooj Mufti

**Affiliations:** 1 Gastroenterology, Mayo Hospital, Lahore, PAK; 2 Internal Medicine, Akbar Niazi Teaching Hospital, Islamabad, PAK; 3 Emergency Medicine, Blackpool Victoria Hospital, Blackpool, GBR; 4 Internal Medicine, Lahore General Hospital, Lahore, PAK; 5 Intensive Care Unit, Epsom and St Helier University Hospitals NHS Trust, Epsom, GBR; 6 Gastroenterology, Indus Hospital, Muzaffargarh, PAK; 7 Nephrology, Lahore General Hospital, Lahore, PAK; 8 Gastroenterology, Lahore General Hospital, Lahore, PAK; 9 Anesthesiology and Critical Care, Mayo Hospital, Lahore, PAK; 10 Cardiology/Medicine, Akhtar Saeed Medical and Dental College, Lahore, PAK

**Keywords:** aggressive hydration, ercp, post-ercp pancreatitis, prophylactic, randomized controlled trial

## Abstract

Background: Post-endoscopic retrograde cholangiopancreatography (ERCP) pancreatitis is one of the important procedure-related complications of ERCP. Periprocedural intravenous hydration with lactated Ringer’s solution has been considered a simple and feasible measure for reducing this complication. The present trial was designed to assess whether aggressive hydration with lactated Ringer’s solution was more effective than standard hydration in preventing post-ERCP pancreatitis (PEP).

Methods: This randomized controlled trial was performed at two tertiary care hospitals. A total of 126 patients undergoing first-time ERCP were included and randomly distributed into two equal treatment groups. Patients in group A were managed with standard hydration, whereas patients in group B received aggressive hydration using lactated Ringer’s solution. The main outcome measure was the development of PEP. Other recorded outcomes were post-ERCP hyperamylasemia, isolated hyperamylasemia, serial pain scores on the visual analog scale, serum amylase levels, duration of hospital stay, and fluid-related adverse effects.

Results: All 126 randomized patients completed the 24-hour assessment. PEP developed in 14 patients in the standard hydration arm and four patients in the aggressive hydration arm (22.2% versus 6.3%; p = 0.020; RR = 0.29, 95% CI = 0.10-0.82). Total post-ERCP hyperamylasemia was less frequent after aggressive hydration than standard hydration (14.3% versus 41.3%; p = 0.001). Isolated hyperamylasemia was recorded in 7.9% and 19.0% of patients, respectively (p = 0.116). Pain scores remained lower with aggressive hydration at 4, 12, and 24 hours (all p < 0.001). Hospital stay was also reduced (1.8 versus 3.0 days; p < 0.001).

Conclusions: Aggressive hydration with lactated Ringer’s solution significantly reduced PEP, post-procedure hyperamylasemia, pain scores, and hospital stay compared with standard hydration. The regimen was well tolerated in selected patients without fluid-overload risk.

## Introduction

Endoscopic retrograde cholangiopancreatography (ERCP) is a therapeutic procedure for pancreaticobiliary diseases, particularly choledocholithiasis, bile duct leak, biliary obstruction, and related obstructive conditions. It remains associated with procedure-related complications, including abdominal pain, bleeding, cholangitis, perforation, and post-ERCP pancreatitis (PEP) [1]. PEP is one of the most frequent clinically important complications and has been reported in up to 10% of ERCP procedures, with higher rates in patients having recognized patient-related and procedure-related risk factors [2]. It is diagnosed when new or worsened pancreatic-type abdominal pain occurs after ERCP with pancreatic enzyme elevation more than three times the upper limit of normal and a need for hospital admission or prolongation of hospital stay. This complication is important because it increases patient discomfort, delays recovery, prolongs admission, raises treatment cost, and may occasionally progress to severe pancreatitis [3,4]. Therefore, prevention of PEP remains an important part of safe ERCP practice.

Different approaches have been used to lower the chance of pancreatitis after ERCP. These include rectal non-steroidal anti-inflammatory drugs, pancreatic duct stent placement in selected high-risk patients, wire-guided cannulation, limiting avoidable pancreatic duct contrast injection, and careful endoscopic technique [4,5]. Periprocedural hydration with lactated Ringer’s solution has also received attention, as adequate circulating volume may help maintain pancreatic perfusion and limit early inflammatory injury after pancreatic irritation. In an early randomized trial, aggressive lactated Ringer’s hydration was associated with fewer cases of pancreatitis after ERCP [6,7]. Reduction in PEP was also described with aggressive hydration in hospitalized patients and in a multicenter randomized trial using lactated Ringer’s solution [8,9]. However, the reported benefit has not been consistent across all studies. Differences in patient risk, hydration schedule, fluid timing, procedural complexity, and outcome definitions have produced variation in results [10,11]. Therefore, routine use of aggressive hydration still requires careful patient selection, monitoring, and assessment of fluid overload risk.

This study was undertaken because aggressive hydration with lactated Ringer’s solution is inexpensive, easily available, and practical during routine ERCP, but its value required assessment in the local setting through a fixed protocol. Comparing it with standard hydration was clinically appropriate, as both approaches can be used during ERCP and their effects can be assessed through post-procedure pain, serum amylase, PEP, isolated hyperamylasemia, and hospital stay. Local randomized evidence on this preventive measure is scarce. Therefore, this trial evaluated the efficacy of aggressive lactated Ringer’s hydration in preventing PEP among eligible patients without fluid-loading contraindications.

## Materials and methods

This randomized controlled trial was carried out in the Gastroenterology Department of Lahore General Hospital (IRB: 221), Lahore, and Mayo Hospital, Lahore (IRB: 178 /RC/KEMU) over a one-year period from July 2024 to June 2025. Permission for the study was obtained from the Institutional Review Board of the institutes. The trial was prospectively entered in the Clinical Trials Registry (NCT07417020). Written informed consent was obtained from all eligible patients before enrolment. Confidentiality was maintained by assigning a study identification number to each participant.

A total of 126 patients were enrolled, with 63 patients in each group. The sample size was calculated by taking the expected frequency of PEP as 22.7% in patients receiving standard hydration and 5.3% in patients receiving aggressive hydration, with a 95% confidence level and 80% power of the test [12]. Eligible patients were recruited by consecutive sampling and were then allocated to either the standard hydration group or the aggressive hydration group by simple randomization using a computer-generated random sequence. Allocation was performed in a 1:1 ratio.

Adults aged 18-70 years, irrespective of gender, were enrolled if they were scheduled for their first ERCP for choledocholithiasis, bile duct leak, or biliary obstruction. Patients were not included if they had undergone ERCP, sphincterotomy, or papillary balloon dilatation previously. Those with chronic pancreatitis, acute pancreatitis, gallstone pancreatitis, or active cholangitis were also excluded. Other exclusion criteria were coagulopathy, ongoing anticoagulant use, pregnancy, breastfeeding, alcohol or substance abuse, hypernatremia or hyponatremia, hyperkalemia, cardiac disease of New York Heart Association class II or higher, respiratory insufficiency with oxygen saturation below 90%, renal impairment with creatinine clearance <40 mL/min, liver dysfunction, peripheral or pulmonary edema, persistent hypotension, sepsis, and planned sphincterotomy during the index ERCP.

Baseline characteristics were documented before ERCP on a predesigned data collection form. These included age, gender, comorbid conditions, indication for ERCP, baseline abdominal pain score measured by the visual analog scale, and baseline serum amylase levels.

Patients allocated to group A received standard hydration with intravenous lactated Ringer’s solution at 1.5 mL/kg/hour during ERCP and for the following eight hours. If PEP developed, these patients received a bolus of 20 mL/kg, followed by lactated Ringer’s solution at 3 mL/kg/hour. Patients allocated to group B received aggressive hydration with intravenous lactated Ringer’s solution at 3 mL/kg/hour during ERCP. Immediately after the procedure, a bolus of 20 mL/kg was administered, followed by lactated Ringer’s solution at 3 mL/kg/hour for the next eight hours. If no abdominal pain was reported after eight hours, the infusion rate was reduced to 1.5 mL/kg/hour. In both groups, hydration was stopped once the patient was able to tolerate a regular oral diet comfortably. Patients were monitored clinically for features of fluid overload, hypotension, respiratory compromise, and other adverse events during the post-procedure period. ERCP was performed as per institutional guidelines uniformly for all patients and according to the indication requirement. 

The primary endpoint was PEP. It was diagnosed when new or increased abdominal pain developed within 24 hours after ERCP, together with pancreatic enzyme elevation to more than three times the upper normal limit at 24 hours, and the patient required admission for at least two days. Hyperamylasemia was recorded when serum amylase exceeded three times the normal value, taken as >300 U/L, during the 24-hour follow-up. Post-procedure pancreatic pain was considered present when epigastric pain radiated to the back with new pain onset, or when pre-existing pain increased by at least three points on the 0-10 visual analog scale and persisted during follow-up. Length of hospital stay was measured in days from the date of ERCP to discharge. Follow-up assessments were performed during the first 24 hours after ERCP and continued until discharge. Serum amylase was measured at 12 and 24 hours after the procedure. Post-procedure pain was assessed using the visual analog scale at 4, 12, and 24 hours. The development of PEP, isolated hyperamylasemia, need for prolonged admission, and length of hospital stay were recorded.

Data were analyzed using SPSS version 26.0 (IBM Corp., Armonk, NY). Quantitative variables such as age, baseline pain score, serum amylase level, post-procedure pain score, and length of hospital stay were summarized as mean ± standard deviation when normally distributed, and as median with interquartile range when distribution was non-normal. Categorical variables such as gender, indication for ERCP, comorbidities, hyperamylasemia, and PEP were presented as frequency and percentage. Normality of quantitative data was assessed before applying comparative tests. The independent sample t-test or Mann-Whitney U test was used for comparison of quantitative variables between the two groups, according to data distribution. The chi-square test or Fisher’s exact test was used for comparison of categorical variables. Repeated pain scores measured at 4, 12, and 24 hours were compared between groups using an appropriate repeated-measures test. A p-value of ≤0.05 was considered statistically significant.

## Results

Both study groups were comparable at baseline, with no statistically significant difference in demographic, clinical, laboratory, or procedural variables. The mean age was 50.6 years in the standard hydration group and 47.9 years in the aggressive hydration group, while female patients were slightly more frequent in the aggressive hydration group. All baseline characteristics also showed no significant difference. Detailed baseline characteristics are presented in Table 1.

**Table 1:** Baseline demographic, clinical, and procedural characteristics of study participants. Data are shown as mean ± SD, median (IQR), or n (%), as appropriate. Between-group comparisons used independent-samples t-test, Mann-Whitney U test, Pearson chi-square test, or Fisher’s exact test. Sphincterotomy refers to the index ERCP procedure. All baseline comparisons were non-significant. ERCP = endoscopic retrograde cholangiopancreatography; BMI = body mass index; IHD = ischemic heart disease; VAS = visual analog scale; IQR = interquartile range; U/L = units per liter.

Variable	Standard hydration (Group A, n = 63)	Aggressive hydration (Group B, n = 63)	Test statistic (df)	p-value
Demographic and anthropometric variables				
Age, years (mean ± SD)	50.6 ± 10.6	47.9 ± 13.3	t = 1.263, df = 124	0.209
Gender - Female, n (%)	35 (55.6%)	40 (63.5%)	χ² = 0.527, df = 1	0.468
Gender - Male, n (%)	28 (44.4%)	23 (36.5%)		
BMI, kg/m² (mean ± SD)	23.8 ± 3.9	24.9 ± 3.6	t = −1.647, df = 124	0.102
Comorbid conditions				
Diabetes mellitus, n (%)	11 (17.5%)	18 (28.6%)	χ² = 1.613, df = 1	0.204
Hypertension, n (%)	21 (33.3%)	27 (42.9%)	χ² = 0.841, df = 1	0.359
Ischemic heart disease, n (%)	11 (17.5%)	11 (17.5%)	χ² < 0.001, df = 1	1.000
ERCP indication				
Choledocholithiasis, n (%)	36 (57.1%)	37 (58.7%)	χ² = 4.887, df = 2	0.087
Biliary obstruction, n (%)	18 (28.6%)	16 (25.4%)		
Bile duct leak, n (%)	9 (14.3%)	10 (15.9%)		
Baseline laboratory parameters				
Serum amylase, U/L (mean ± SD)	83.2 ± 20.5	76.4 ± 22.6	t = 1.764, df = 124	0.080
Serum lipase, U/L (mean ± SD)	53.7 ± 11.5	51.8 ± 12.6	t = 0.876, df = 124	0.383
Baseline VAS score - median (IQR)	1.4 (0.5-1.9)	1.3 (0.8-1.9)	U = 2014	0.887
Procedural variables				
Procedure duration, min - median (IQR)	38.0 (32.0-43.5)	35.0 (29.5-45.0)	U = 2100	0.576
Sphincterotomy performed during index ERCP, n (%)	59 (93.7%)	57 (90.5%)	χ² = 0.109, df = 1	0.742
Pancreatic duct cannulation, n (%)	13 (20.6%)	11 (17.5%)	χ² = 0.051, df = 1	0.821

**Figure 1:**
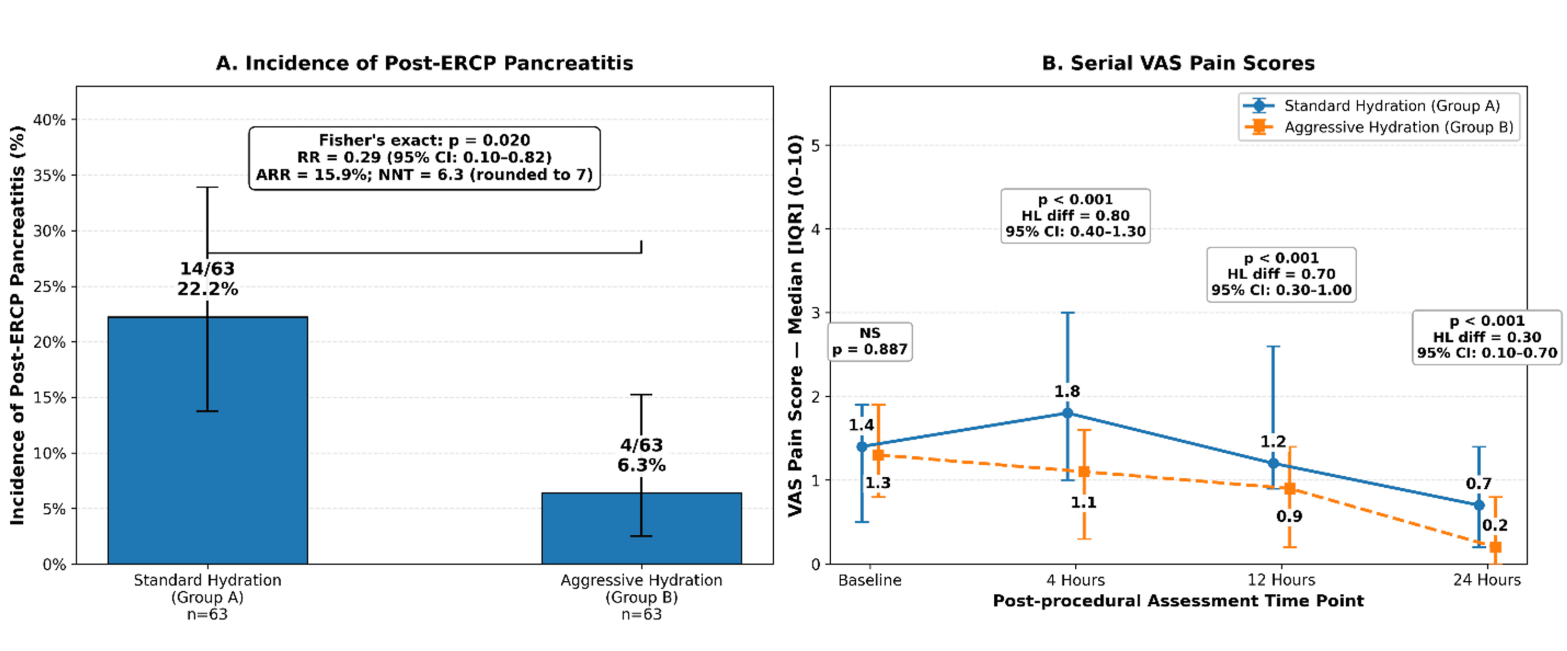
Primary outcome and serial pain assessment by hydration group. (A) Incidence of post-ERCP pancreatitis in the standard hydration and aggressive hydration groups, with error bars representing 95% confidence intervals for proportions. (B) Serial VAS pain scores at baseline, 4 hours, 12 hours, and 24 hours after ERCP, reported as median (IQR). Error bars in panel B represent interquartile ranges. Between-group VAS comparisons were performed using the Mann-Whitney U test with Hodges-Lehmann median difference estimates. ERCP = endoscopic retrograde cholangiopancreatography; PEP = post-ERCP pancreatitis; VAS = visual analog scale; RR = relative risk; CI = confidence interval; ARR = absolute risk reduction; NNT = number needed to treat; HL diff = Hodges-Lehmann median difference; IQR = interquartile range.

PEP developed in 14/63 patients in group A and 4/63 patients in group B, corresponding to 22.2% and 6.3%, respectively. Fisher’s exact test showed a significant difference between groups (p = 0.020). The estimated relative risk was 0.29 (95% CI = 0.10-0.82), while the absolute risk reduction was 15.9%. The number needed to treat was calculated as 6.3 and rounded to 7. In group A, PEP severity was mild in seven patients, moderate in five, and severe in two patients. In group B, three patients had mild disease, and one had moderate disease. No severe PEP episode occurred in group B. These data are presented in Table 2 and Figure 1.

**Table 2:** Primary and secondary outcomes: standard vs. aggressive hydration with lactated Ringer's solution. PEP severity was graded according to the Cotton consensus criteria. ‡ Fisher’s exact test. Repeated-measures variables were analyzed using the Friedman test. Statistically significant p-values were defined as p < 0.05. ERCP = endoscopic retrograde cholangiopancreatography; PEP = post-ERCP pancreatitis; RR = relative risk; ARR = absolute risk reduction; NNT = number needed to treat; HL diff = Hodges-Lehmann median difference; CI = confidence interval; VAS = visual analog scale; IQR = interquartile range; LOS = length of hospital stay; ULN = upper limit of normal for serum amylase, taken as 130 U/L; 3× ULN = 390 U/L.

Outcome variable	Standard hydration (Group A, n = 63)	Aggressive hydration (Group B, n = 63)	Effect estimate (95% CI)	p-value
PEP, n (%)	14 (22.2%)	4 (6.3%)	RR = 0.29 (95% CI = 0.10-0.82)	0.020
Mild PEP, n (%)	7 (11.1%)	3 (4.8%)		
Moderate PEP, n (%)	5 (7.9%)	1 (1.6%)		
Severe PEP, n (%)	2 (3.2%)	0 (0.0%)		
Absolute risk reduction (ARR)			ARR = 15.9%	—
Number needed to treat (NNT)			NNT = 6.3 (≈7)	—
Total post-ERCP hyperamylasemia, n (%)	26 (41.3%)	9 (14.3%)	RR = 0.35 (95% CI = 0.18-0.68)	0.001
Concurrent with PEP (amylase >390 + pain ≥3), n (%)	14 (22.2%)	4 (6.3%)	(Same as primary outcome)	0.020
Isolated hyperamylasemia (amylase >390, pain <3), n (%)	12 (19.0%)	5 (7.9%)	RR = 0.42 (95% CI = 0.16-1.07)	0.116
VAS at baseline (pre-procedural)	1.4 (0.5-1.9)	1.3 (0.8-1.9)	HL diff = 0.00 (95% CI = −0.40 to 0.30)	0.887
VAS at 4 hours post-ERCP	1.8 (1.0-3.0)	1.1 (0.3-1.6)	HL diff = 0.80 (95% CI = 0.40-1.30)	< 0.001
VAS at 12 hours post-ERCP	1.2 (0.9-2.6)	0.9 (0.2-1.4)	HL diff = 0.70 (95% CI = 0.30-1.00)	< 0.001
VAS at 24 Hours post-ERCP	0.7 (0.2-1.4)	0.2 (0.0-0.8)	HL diff = 0.30 (95% CI = 0.10-0.70)	< 0.001
Repeated-measures (group A)			χ² = 28.64	< 0.001
Repeated-measures (group B)			χ² = 27.35	< 0.001
Serum amylase baseline	83.8 (69.7-97.5)	75.6 (60.6-89.8)	HL diff = 7.00 U/L (95% CI = −0.20-15.26)	0.071
Serum amylase at 12 hours post-ERCP	128.9 (82.6-451.9)	62.3 (34.9-91.7)	HL diff = 60.2 U/L (95% CI = 40.2-97.0)	< 0.001
Serum amylase at 24 hours post-ERCP	99.6 (68.5-422.9)	57.7 (40.3-73.8)	HL diff = 46.0 U/L (95% CI = 28.9-83.2)	< 0.001
Repeated-measures (group A)			χ² = 15.83	< 0.001
Repeated-measures (group B)			χ² = 16.44	< 0.001
Length of stay - median (IQR), days	3.0 (2.3-3.8)	1.8 (1.4-2.4)	HL diff = 1.10 days (95% CI = 0.70-1.50)	< 0.001
LOS - PEP patients only, median (IQR)	6.3 (5.6-7.7) (n = 14)	4.1 (3.3-5.1) (n = 4)	—	—
LOS - Non-PEP patients, median (IQR)	2.7 (2.3-3.2) (n = 49)	1.8 (1.4-2.2) (n = 59)	—	—
Volume overload (peripheral edema), n (%)	0 (0.0%)	2 (3.2%)	—	0.497

Post-procedure hyperamylasemia was found in 26/63 patients in group A compared with 9/63 patients in group B (41.3% versus 14.3%; p = 0.001; RR = 0.35, 95% CI = 0.18-0.68). Isolated enzyme rises without the pain criterion occurred in 12 patients in group A and five patients in group B (19.0% versus 7.9%), with no significant difference (p = 0.116). Baseline VAS scores were not different between groups (p = 0.887). After ERCP, pain scores were lower in group B at 4, 12, and 24 hours (all p < 0.001). Friedman's analysis showed significant within-group change in serial VAS scores in both groups. These findings are summarized in Table 2 and Figure 1.

Serum amylase before ERCP was comparable between the two groups (p = 0.071). At 12 and 24 hours, amylase levels were significantly higher in group A than in group B (both p < 0.001), as shown in Table 2. Median hospital stay was longer in group A than in group B (3.0 versus 1.8 days; p < 0.001). Mild peripheral edema was recorded in two patients in group B. No patient developed pulmonary edema, oxygen desaturation, hemodynamic compromise, required diuretics, or needed discontinuation of the hydration protocol.

## Discussion

The present randomized trial found a lower frequency of PEP in patients receiving aggressive lactated Ringer’s hydration compared with standard hydration. The absolute risk reduction and number needed to treat indicate a clinically meaningful difference, while the lower post-procedure amylase levels, reduced VAS pain scores, and shorter hospital stay support the primary outcome pattern without adding major fluid-related safety concerns.

The reduction in PEP in the aggressive hydration group is in close agreement with trials that used a similar lactated Ringer’s protocol. The results matched a trial showing PEP reduction from 22.7% to 5.3%, using the same hydration and diagnostic definition protocol [12]. A comparable pattern was seen in a small pilot trial, but the absence of PEP in the aggressive hydration group should be read carefully because the sample was limited and group allocation was unequal [13]. The lower PEP rates in these trials may reflect differences in protocol design, including pre-procedure bolus administration and separate fluid arms for lactated Ringer’s solution and normal saline, which allowed clearer control of fluid type and timing [8,9]. The present findings differ from the FLUYT trial because all patients in that trial received rectal non-steroidal anti-inflammatory drug (NSAID) prophylaxis, leaving less residual risk for hydration to reduce [14]. The trial using albumin with lactated Ringer’s solution did not show added benefit, indicating that colloid supplementation may not improve PEP prevention when sufficient balanced crystalloid hydration is already given [15]. No severe PEP was seen in the aggressive hydration group, but this should be read carefully because the total number of PEP cases was small. Earlier trials also showed fewer moderate or severe cases with intensive lactated Ringer’s hydration. Studies comparing two active aggressive fluid regimens or albumin-added hydration were less able to show severity differences [8,10,16].

Lower total hyperamylasemia and lower serial serum amylase in the aggressive hydration group supported the primary outcome at the biochemical level, but enzyme rise alone was not treated as clinical pancreatitis. This distinction was important because amylase may increase after ERCP without sustained pain or fulfillment of PEP criteria. The present pattern was in keeping with earlier reports showing fewer enzyme elevations after aggressive hydration, including reductions in hyperamylasemia and lower post-procedure enzyme profiles [11,12,17]. In one previous trial, hyperamylasemia did not differ clearly between groups, although PEP was lower with aggressive lactated Ringer’s hydration. This shows that raised amylase and clinical pancreatitis do not always follow the same pattern [9]. In the present study, isolated hyperamylasemia was also not significant, which is understandable because patients who met full PEP criteria were excluded from this subgroup, leaving fewer cases for comparison. Pooled evidence still supports fewer enzyme rises with aggressive hydration [18,19].

Post-procedure pain remained lower with aggressive hydration, while baseline VAS scores were similar in both groups. This shows that the difference appeared after ERCP and was not due to unequal pain before the procedure. This finding is relevant because abdominal pain is part of PEP diagnosis and may affect observation, fasting, analgesic use, and discharge. Similar reductions in pain were reported with comparable lactated Ringer’s protocols, while the pilot trial had limited power to show a clear difference [11,13,20,21]. Hospital stay was shorter in the aggressive hydration group, which was likely related to fewer PEP events, lower pain scores, and lower enzyme elevation under a single-center discharge pathway, while pooled trials showed variable stay results because discharge rules differed across centers [11,18,19].

Fluid-related adverse events were limited to mild peripheral edema, with no pulmonary edema, oxygen desaturation, hemodynamic compromise, diuretic requirement, or protocol discontinuation. This safety profile is similar to previous trials in which clinically important fluid overload was uncommon after excluding patients with cardiac, renal, hepatic, respiratory, or baseline fluid-overload risk [8,11,13]. The FLUYT and tailored hydration trials also did not show a significant excess of hydration-related complications, although their prophylaxis context differed because of routine rectal NSAID use or adaptive fluid continuation [14]. Pooled evidence supports short-term safety in selected patients, not in those with major cardiopulmonary or renal compromise [18,19].

Lower PEP, pain scores, and serum amylase in the aggressive hydration group can be explained by the changes that occur during ERCP. Papillary trauma, duct handling, and contrast injection can increase pressure inside the pancreatic duct, cause local swelling, and lead to early leakage of pancreatic enzymes. Good fluid support during this period may help maintain pancreatic blood flow and reduce blood concentration. Lactated Ringer’s solution is also less likely than normal saline to cause chloride-related acidosis when larger volumes are used [13,22,23].

Recent trials show that the effect of aggressive hydration depends on what other preventive measures are already being used. When rectal NSAIDs were given to all patients, adding aggressive hydration did not further reduce PEP, probably because the baseline risk had already been lowered [14]. In the present study, NSAID use was not standardized, which may explain the larger difference between groups. Adding albumin to lactated Ringer’s did not reduce PEP or hyperamylasemia, showing that albumin addition is not necessarily better than lactated Ringer’s alone [15]. Tailored hydration based on early pain and enzyme testing also supports careful fluid adjustment after ERCP [20]. Aggressive hydration should therefore be used as an adjunct to rectal NSAIDs, careful cannulation, and pancreatic stenting when indicated.

Strengths of the study included randomized design, equal group allocation, standardized weight-based lactated Ringer’s protocol, clear PEP definition, Cotton severity grading, serial VAS pain assessment, serial serum amylase testing, complete 24-hour follow-up, intention-to-treat analysis, safety monitoring, and appropriate use of non-parametric statistics. Limitations of the study included a single-center setting, modest sample size, short follow-up, no long-term readmission data, no standardized rectal NSAID protocol, limited subgroup analysis, inability to assess rare fluid-related adverse events, and limited applicability to patients with cardiac, renal, hepatic, or fluid-overload risk. Future studies should include larger multicenter trials, longer follow-up, routine recording of rectal NSAID and pancreatic stent use, readmission assessment, cost analysis, and evaluation of tailored hydration based on early pain and enzyme response.

## Conclusions

In this randomized controlled trial, aggressive hydration with lactated Ringer’s solution was more effective than standard hydration in preventing PEP among eligible patients undergoing first-time ERCP. It was also associated with lower post-procedure hyperamylasemia, reduced abdominal pain during early assessment, and shorter hospital stay. The treatment was generally tolerated, with no serious fluid-related cardiopulmonary complications observed. These findings support aggressive lactated Ringer’s hydration as a preventive adjunct in appropriate patients, provided that contraindications to fluid loading are excluded and post-procedure clinical monitoring is maintained.
